# Fetal head circumference, operative delivery, and fetal outcomes: a multi-ethnic population-based cohort study

**DOI:** 10.1186/1471-2393-13-106

**Published:** 2013-05-07

**Authors:** Andrew Mujugira, Alfred Osoti, Ruth Deya, Stephen E Hawes, Amanda I Phipps

**Affiliations:** 1 Department of Epidemiology, University of Washington, Seattle, WA, USA; 2 Fred Hutchinson Cancer Research Center, Seattle, WA, USA; 3 International Clinical Research Center, Department of Global Health, University of Washington, 325 Ninth Avenue, Box 359927, Seattle, WA, 98104, USA

**Keywords:** Fetal head circumference, Cesarean section, Operative delivery, Fetal distress, Low Apgar score

## Abstract

**Background:**

Operative delivery procedures, such as primary cesarean section, vacuum-assisted, and forceps-assisted vaginal delivery increase maternal and fetal morbidity, and the cost of care. We evaluated whether large fetal head circumference (FHC) independently increases risk of such interventions, as well as fetal distress or low Apgar score, in anatomically normal infants.

**Methods:**

We conducted a population-based retrospective cohort study using Washington State birth certificate data. We included singleton, term infants born to nulliparous mothers from 2003–2009. We compared mode of delivery and fetal outcomes in 10,750 large-FHC (37-41 cm) infants relative to 10,750 average-FHC (34 cm) infants, frequency matched by birth-year.

**Results:**

Large-FHC infants were nearly twice as likely to be delivered by primary cesarean section as average-FHC infants (unadjusted relative risk [RR] 1.84, 95% confidence interval [CI]: 1.77, 1.92). The RR for primary cesarean section associated with large-FHC was largest for mothers aged 19 years or less (RR 2.28; 95% CI: 1.99, 2.61), and smallest for mothers aged 35 years or greater (RR 1.51; 95% CI: 1.37, 1.66) [test of homogeneity, p < 0.001]. Large-FHC infants were at increased risk of vacuum-assisted vaginal delivery (RR 1.55; 95% CI: 1.43, 1.69), and forceps-assisted vaginal delivery (RR 1.61; 95% CI: 1.32, 1.97). There was no difference in risk of fetal distress (RR 0.97; 95% CI: 0.89, 1.07) for large-FHC versus average-FHC infants. Risk estimates were unaffected by adjustment for potential confounders.

**Conclusions:**

Nulliparous mothers of large-FHC infants are at increased risk of primary cesarean section, vacuum-assisted and forceps-assisted vaginal delivery relative to mothers of average-FHC infants. Maternal age modifies the association between FHC and primary cesarean section.

## Background

Operative delivery procedures arising from prolonged labor increase maternal morbidity, fetal morbidity, and the cost of care. Cephalopelvic disproportion (CPD), due to narrow maternal pelvic diameter relative to fetal head circumference (FHC) or large FHC relative to maternal pelvic diameter, is the main cause of prolonged labor [1]. In the United States, one third of all deliveries in 2009 were by cesarean section, a record high [2]. Since 1996, cesarean delivery rates have increased by more than 50% among all maternal age groups [3]; up to 50% of this increase has been attributed to primary cesarean sections. These increases carry significant public health impact, because cesarean deliveries are associated with increased risk of maternal and fetal morbidity and mortality [4], and cost almost twice as much as uncomplicated vaginal deliveries [5].

Operative vaginal delivery [6] and large FHC [7] are associated with a 3–5 fold increased risk of pelvic floor trauma. Pelvic floor trauma during vaginal delivery is independently associated with pelvic floor disorders (e.g. stress urinary incontinence and pelvic organ prolapse) [8]. A recent population-based Swedish study that evaluated postnatal head circumference suggested that women delivering infants with large FHC (39-41 cm) were significantly more likely to experience vacuum-assisted vaginal delivery, fetal distress, prolonged labor and cesarean section relative to women delivering infants with average FHC [9]. Among nulliparous Irish women, large FHC was significantly associated with prolonged labor with similar sensitivity and specificity as elevated birth weight [10].

The potential of FHC in predicting risk of operative delivery (i.e., cesarean section, vacuum-assisted, or forceps-assisted vaginal delivery), fetal distress and low Apgar score in anatomically normal fetuses and ethnically diverse populations has not been previously examined. Understanding the association between FHC, and adverse maternal and fetal outcomes is essential in planning intrapartum care, including neonatal resuscitation. Ultrasound FHC measurements at term, as an independent and prenatal risk factor for operative delivery, may guide the timing of cesarean sections [11]. Identifying pregnant women at greatest risk of pelvic floor disorders following vaginal delivery may inform modification of intrapartum procedures.

The purpose of this study was to determine the risk of primary cesarean section and assisted vaginal delivery among mothers of large FHC infants (≥37 cm) relative to mothers of average FHC infants (34 cm), and risk of fetal distress and low Apgar score among large FHC infants relative to average FHC infants, in the ethnically diverse population of Washington State, USA.

## Methods

### Subjects and setting

We conducted a population-based retrospective cohort study using birth certificate data from the Washington State Department of Health for infants born between 2003 and 2009. We used newborn head circumference as a proxy measure of FHC, given the strong correlation between sonographic and postnatal measurements of head circumference [11]. Head circumference was measured from the occiput to the supraorbital ridges using a flexible non-stretchable measuring tape. Infants were classified as having a large FHC if they had a postnatal head circumference of 37-41 cm (i.e., ≥97 th percentile for the United States population) measured immediately after birth. These exposed infants were compared to an equal number of randomly selected infants with an average FHC, defined as a postnatal head circumference of 34 cm (50 th percentile), and frequency matched by year of birth. Similar head circumference cutoffs were used in prior studies [6,10].

We limited our study population to nulliparous women with singleton pregnancies in cephalic presentation to exclude pre-planned indications of operative delivery. Inclusion was also limited to term pregnancies, defined as a gestational age of 37–42 weeks using the estimated date of last normal menstrual period. We excluded infants with congenital malformations, and infants with low birth weight (<2500 grams) because these infants are at increased risk of cesarean section and fetal distress. In addition, we excluded mothers from the study population with diabetes and third trimester obstetric hemorrhage, as both conditions are associated with increased risk of operative delivery.

Information on the modes of delivery and fetal outcomes were obtained from birth certificate records. The primary outcomes of interest included primary cesarean section, vacuum-assisted vaginal delivery, forceps-assisted vaginal delivery, clinical diagnosis of fetal distress or non-reassuring fetal status (based on intrapartum signs of stress including abnormal fetal heart rate patterns, or biochemical indicators of fetal distress and/or meconium staining of amniotic fluid) [12], and low Apgar score (defined as 5-minute APGAR scores of less than 4, indicative of an increased risk of poor neurologic outcomes) [13]. All outcomes were ascertained from the birth certificates. We identified a total of 10,750 large FHC infants and assessed 10,750 average FHC infants for comparison.

### Statistical analysis

The relative risk of operative delivery outcomes (i.e., primary cesarean section, vacuum-assisted, or forceps-assisted vaginal delivery) among mothers whose infants had large FHC relative to those with average FHC was estimated using stratified analysis by Mantel-Haenszel methods with 95% confidence intervals. We used stratified analysis instead of logistic regression, to examine data within individual strata, compute stratum-specific risk estimates adjusted for each confounder, and identify effect modification. We assessed effect modification by considering biologic plausibility, the magnitude and direction of differences in stratum specific risk estimates, and tests of homogeneity. We evaluated maternal age (≤19, 20–24, 25–29, 30–34, ≥35 years) and infant sex as potential effect modifiers since risk of operative delivery differs with age [14], and males have, on average, larger FHC than females [15]. In the absence of effect modification, maternal age and infant sex were evaluated as potential confounders. In addition, we considered the following as potential confounders: race/ethnicity (non-Hispanic White, Black, Hispanic, Asian, Native American, and Pacific Islander), pre-pregnancy body mass index (BMI, computed from maternal weight and height on the birth certificate) (≤18.5, 19–24.9, 25–29, 30–34, ≥35 kg/m^2^) [16,17], birth weight (2500–3500, 3501–4500, >4500 grams), hypertension, preeclampsia, smoking, and epidural analgesia. We excluded prolonged labor and labor induction because they are in the causal pathway between FHC and operative delivery. To estimate the independent effect of FHC on risk of primary cesarean section, we adjusted for birth weight in sensitivity analyses. Variables that changed the relative risk estimates by at least 10% were included in the final adjusted analysis as confounders. Records with missing values were excluded from the analysis with the exception of ethnicity; mothers with missing ethnicity (n = 194) were classified as unknown. Statistical analyses were performed using Stata 12.1 (StataCorp, College Station, TX). The Institutional Review Board of the University of Washington approved this study.

## Results

Relative to mothers of average FHC infants, mothers of large FHC infants were more likely to be aged 30 years or greater (30% vs. 25%), white (82% vs. 74%), obese (23% vs. 17%), and to have had labor induction (35% vs. 24%) (Pearson Chi-square p < 0.001 for all comparisons). Compared to average FHC infants, large FHC infants were more likely to be male (70% vs. 50%) and were more likely to have a birth weight greater than 3500 grams (86% vs. 40%) (Table 1).

**Table 1:** Maternal and fetal characteristics of infants with large and average fetal head circumference, Washington State, 2003 – 2009

	**Large FHC [≥37 cm]**		**Average FHC [34 cm]**	
	**(N = 10,750)**		**(N = 10,750)**	
Median (interquartile range) or N (%)				
**Maternal characteristics**				
Age (years)	26	(22, 31)	25	(20, 30)
≤19	1371	(13)	1948	(18)
20-24	3086	(29)	3330	(31)
25-29	3039	(28)	2779	(26)
30-34	2152	(20)	1811	(17)
≥35	1099	(10)	878	(8)
Ethnicity				
White	8777	(82)	7985	(74)
Black	347	(3)	439	(4)
Hispanic	585	(5)	943	(9)
Asian	650	(6)	966	(9)
Native American	208	(2)	204	(2)
Pacific Islander	90	(1)	112	(1)
Unknown^a^	93	(1)	101	(1)
BMI^b^				
<18.5	231	(2)	425	(4)
18.5-24.9	4733	(48)	5498	(56)
25-29.9	2631	(27)	2261	(23)
30-34.9	1261	(13)	991	(10)
35+	1050	(10)	629	(7)
Marital status				
Married	7190	(67)	6548	(61)
Single	3545	(33)	4186	(39)
Hypertension (yes)	101	(1)	89	(1)
Induction of Labor (yes)	3719	(35)	2597	(24)
Prolonged labor^c^ (yes)	678	(6)	441	(4)
Smoking (yes)	846	(8)	1058	(10)
Pre-eclampsia (yes)	681	(6)	692	(6)
**Infant characteristics**				
Male sex	7521	(70)	5374	(50)
Birth weight (grams)				
2500-3500	1506	(14)	6388	(59)
3501-4500	8143	(76)	4317	(40)
>4500	1100	(10)	44	(<1)
5 min Apgar scores				
7-10	10518	(98)	10555	(98)
4-6	187	(>1)	167	(>1)
0-3	45	(<1)	28	(<1)

^a^ 194 mothers refused to state their ethnicity. ^b^ 1790 of 21500 (8%) had missing values. ^c^ 99 missing values.

Associations between large FHC and all five outcomes (primary cesarean section, vacuum-assisted vaginal delivery, forceps-assisted vaginal delivery, low Apgar score and fetal distress) were unaffected by adjustment for race/ethnicity, BMI, hypertension, preeclampsia, smoking, labor induction, epidural analgesia, and infant sex; thus, unadjusted risk estimates are presented.

### Operative delivery

We found that large FHC infants were nearly twice as likely to be delivered by primary cesarean section as average FHC infants (Table 2). Notably, the elevated risk of primary cesarean section associated with large FHC decreased with increasing maternal age (test of homogeneity, p < 0.001). Among young mothers (aged 19 years or less), those with large FHC infants had a 2.28-fold (95% confidence interval [CI]: 1.99, 2.61) increased risk of primary cesarean section, whereas, among mothers aged 35 years or greater, having a large FHC infant was associated with a relative risk (RR) of 1.51 (95% CI: 1.37, 1.66) for primary cesarean section. The association between large FHC and primary cesarean section was not modified by infant sex (test of homogeneity, p = 0.267).

**Table 2:** Risk of operative delivery, fetal distress & low Apgar score in infants with large fetal head circumference relative to average head circumference, Washington State 2003-2009

**Outcomes**	**Large FHC**		**Average FHC**		**Unadjusted risk ratio for outcome in large vs. average FHC (95% CI)**
	**(N = 10,750)**		**(N = 10,750)**		
	**n**	**(%)**	**n**	**(%)**	
**Adverse birth outcomes**					
Unassisted vaginal delivery	5160	(48)	7271	(68)	1.00 (Ref)
Vacuum extraction	1067	(10)	901	(8)	1.55 (1.43, 1.69)
Forceps delivery	196	(2)	169	(2)	1.61 (1.32, 1.97)
Primary C-section	4283	(40)	2379	(22)	1.84 (1.77, 1.92)
**Primary cesarean section**^a^					
**Maternal age**					
**≤ 19 years**					
Unassisted vaginal delivery	798	(66)	1490	(85)	1.00 (Ref)
Primary C-section	416	(34)	264	(15)	2.28 (1.99, 2.61)
**20-24 years**					
Unassisted vaginal delivery	1702	(62)	2413	(80)	1.00 (Ref)
Primary C-section	1047	(38)	621	(20)	1.86 (1.71, 2.03)
**25-29 years**					
Unassisted vaginal delivery	1443	(54)	1854	(75)	1.00 (Ref)
Primary C-section	1222	(46)	627	(25)	1.81 (1.68, 1.96)
**30-34 years**					
Unassisted vaginal delivery	876	(47)	1067	(67)	1.00 (Ref)
Primary C-section	993	(53)	536	(33)	1.59 (1.47, 1.72)
**35+ years**					
Unassisted vaginal delivery	340	(36)	445	(54)	1.00 (Ref)
Primary C-section	604	(64)	329	(46)	1.51 (1.37, 1.66)
**Fetal outcomes**					
No fetal distress	9919	(92)	9906	(92)	1.00 (Ref)
Fetal distress^b^	813	(8)	835	(8)	0.97 (0.89, 1.07)
No low Apgar score	10696	(99)	10714	(99)	1.00 (Ref)
Low Apgar score ^c^	45	(<1)	28	(<1)	1.61 (1.00, 2.57)

^a^ Excludes 63 non-primary C-sections ^b^ 27 missing values ^c^ 17 missing values.

In sensitivity analyses, the increased risk of cesarean delivery associated with large FHC was modestly attenuated after adjusting for birth weight, but remained statistically significant (aRR 1.57; 95% CI: 1.50, 1.65) suggesting an independent effect of FHC on risk of primary cesarean section over and above that of birth weight. This increased risk was observed in all ethnic groups.

Risk of vacuum-assisted vaginal delivery was also significantly elevated for large FHC infants relative to average FHC infants (RR 1.55; 95% CI: 1.43, 1.69). Similarly, infants with large FHC were at increased risk of forceps-assisted vaginal delivery (RR 1.61; 95% CI: 1.32, 1.97). The effect of large FHC on vacuum or forceps-assisted vaginal delivery did not vary by maternal age or infant sex.

### Fetal outcomes

The increased risk of low Apgar score in infants with large FHC was of borderline statistical significance (RR 1.61; 95% CI: 1.00, 2.57). We observed no difference in risk of fetal distress (RR 1.06; 95% CI: 0.95, 1.18) according to FHC. We did not find evidence that the association between large FHC and these two outcomes (fetal distress and low Apgar score) was modified by infant sex (test of homogeneity, p = 0.69 and 0.32, respectively).

## Discussion

In this population-based study of 21,500 mother-infant pairs, mothers of large FHC infants were at increased risk of operative delivery (primary cesarean section, vacuum-assisted and forceps-assisted vaginal delivery) compared to mothers of average FHC infants. Nulliparous mothers of large FHC infants had an almost two-fold increased risk of primary cesarean section; this increased risk was attenuated with increasing maternal age.

A small number of previous studies have evaluated the relationship between large FHC, operative delivery and fetal outcomes [9,10]. A large Swedish study that used similar FHC definitions to our study, evaluated the influence of large FHC on labor outcomes [9]. In that study, nulliparous mothers with large FHC infants had elevated risk of both vacuum-assisted vaginal delivery (odds ratio [OR] 3.47; 95% CI: 3.10, 3.88) and cesarean delivery (OR 1.22; 95% CI: 1.04, 1.42). Although FHC was associated with both of these outcomes in our study as well, we observed a more modest association with risk of vacuum-assisted vaginal delivery and a stronger association with risk of primary cesarean delivery. These findings may reflect differences in operative delivery management between Sweden and the United States.

To our knowledge, this is the first study to report effect modification, on the multiplicative scale, by maternal age in the relationship between large FHC and primary cesarean section. A nationally representative study of teenage mothers aged 12–20 years found that cesarean delivery rates decreased with increasing maternal age, and that this effect was modified by macrosomia (i.e., birth weight >4000 g) [18]. Younger maternal age is associated with skeletal immaturity, including smaller bony pelvises [18]. A study from Finland found that during the transition from adolescence to early adulthood, pelvic width increased with age at a slower rate than height. Consequently by age 18, girls had attained their mother’s height but not pelvic width [19]. We hypothesize that pelvic inadequacy in younger nulliparae with large FHC infants (i.e., cephalopelvic disproportion [CPD]) elevates risk of primary cesarean delivery, which modestly decreases as pelvic maturity is attained. Although CPD is the most common indication for primary cesarean delivery in nulliparous mothers irrespective of age, younger mothers are at higher risk of cesarean delivery [20]. A systematic review of adolescent pregnancies in the United States reported a four-fold increase in cesarean delivery rates over a 25-year period [21]. Moreover, the greatest increase in cesarean deliveries (57%) in the United States between 2000 and 2007 was among women aged 25 years or less [3]. These studies suggest elevated risk of cesarean delivery at younger maternal ages. Our results are consistent with these findings.

Approximately 1 in 25 births in the United States are vacuum-assisted or forceps-assisted vaginal deliveries [2]. Vaginal-assisted delivery is commonly indicated for prolonged labor, but contraindicated for CPD [22]. We found that nulliparous mothers of large FHC infants were significantly more likely to experience prolonged labor relative to mothers of average FHC infants (p < 0.001), which may have increased risk of assisted vaginal delivery.

Our study strengths include the large sample size and diversity of a multi-ethnic cohort. Our study has limitations. We did not have hospital discharge data; thus, it is possible that some instances of operative delivery and adverse fetal outcomes were missed. However, the accuracy of birth certificate data alone is comparable with combined data for vacuum-assisted and forceps-assisted vaginal deliveries [23]. Therefore, it is unlikely that the lack of hospital discharge data significantly impacts our findings. Although the Washington State birth certificate check-box format permits more accurate reporting [24], we did have to exclude some individuals with missing FHC data from the eligible study population; however, the proportion of records with missing FHC was not substantial (0.3%). Head circumference was measured soon after birth, but does not differ markedly from intra-partum FHC [11]. CPD may have contributed to the associations we observed. However, because pelvimetry was not evaluated in our study population, we were not able to determine the role of CPD on risk of operative delivery or fetal outcomes. Nevertheless, our findings remain valid and generalizable since pelvic diameters are not routinely measured during pregnancy. Indications for operative delivery were not recorded on the birth certificate; such information could further elucidate our observed findings and their clinical utility. Although the majority of women with large FHC infants successfully delivered vaginally, knowledge of FHC may be helpful in triage of care so that women with large FHC can access facilities that are able to offer emergency cesarean section especially if they are younger nulliparae. We did not have information on laboratory records of academia or neonatal pH. Therefore we were not able to make a diagnosis of low Apgar score. However the clinical decision-making process and management are based on clinical diagnosis of low APGAR score [12,25]. In addition, we did not distinguish between elective and emergency primary cesarean sections, and onset of labor may have confounded our results.

## Conclusions

We found that large FHC increased the risk of primary cesarean section and assisted vaginal delivery in a large ethnically diverse population. The association between large FHC and primary cesarean section was modified by maternal age.

Future prospective studies should evaluate the utility of pre-induction or early labor ultrasound measurements of FHC in predicting risk of operative delivery to address the public health question of whether screening programs using FHC as a predictor for operative delivery should target limited resources to younger nulliparae.

## Competing interests

The authors declare that they have no competing interests.

## Authors’ contributions

SH, RD, AO and AM designed the study. AM and AIP wrote the first draft. AO, RD and AM performed the statistical analyses. All authors contributed to the interpretation of the results and the writing of the manuscript, and all approved the final draft.

## Pre-publication history

The pre-publication history for this paper can be accessed here:

http://www.biomedcentral.com/1471-2393/13/106/prepub

## References

[B1] Konje JC Ladipo OA Nutrition and obstructed labor Am J Clin Nutr 2000 72 1 Suppl 291S 297S 10871595 10.1093/ajcn/72.1.291S

[B2] Martin JA Hamilton BE Ventura SJ Osterman MJ Kirmeyer S Matthews TJ Wilson EC Births: final data for 2009 Natl Vital Stat Rep 2011 60 1 1 14 22670489

[B3] Menacker F Hamilton B Recent trends in cesarean delivery in the united states. In., vol. No. 35 2010 Atlanta: National Center for Health Statistics

[B4] Villar J Valladares E Wojdyla D Zavaleta N Carroli G Velazco A Shah A Campodonico L Bataglia V Faundes A Caesarean delivery rates and pregnancy outcomes: the 2005 WHO global survey on maternal and perinatal health in Latin America Lancet 2006 367 9525 1819 1829 10.1016/S0140-6736(06)68704-7 16753484

[B5] Hospitalizations related to childbirth http://www.hcup-us.ahrq.gov/reports/statbriefs/sb110.jsp

[B6] Bahl R Strachan B Murphy DJ Pelvic floor morbidity at 3 years after instrumental delivery and cesarean delivery in the second stage of labor and the impact of a subsequent delivery Am J Obstet Gynecol 2005 192 3 789 794 10.1016/j.ajog.2004.10.601 15746673

[B7] Valsky DV Lipschuetz M Bord A Eldar I Messing B Hochner-Celnikier D Lavy Y Cohen SM Yagel S Fetal head circumference and length of second stage of labor are risk factors for levator ani muscle injury, diagnosed by 3-dimensional transperineal ultrasound in primiparous women Am J Obstet Gynecol 2009 201 1 91 e91-97 19481726 10.1016/j.ajog.2009.03.028

[B8] Lukacz ES Lawrence JM Contreras R Nager CW Luber KM Parity, mode of delivery, and pelvic floor disorders Obstet Gynecol 2006 107 6 1253 1260 10.1097/01.AOG.0000218096.54169.34 16738149

[B9] Elvander C Hogberg U Ekeus C The influence of fetal head circumference on labor outcome: a population-based register study Acta Obstet Gynecol Scand 2012 91 4 470 475 10.1111/j.1600-0412.2012.01358.x 22229662

[B10] Kennelly MM Anjum R Lyons S Burke G Postpartum fetal head circumference and its influence on labour duration in nullipara J Obstet Gynaecol 2003 23 5 496 499 10.1080/0144361031000153701 12963505

[B11] Melamed N Yogev Y Danon D Mashiach R Meizner I Ben-Haroush A Sonographic estimation of fetal head circumference: how accurate are we? Ultrasound Obstet Gynecol 2011 37 1 65 71 10.1002/uog.7760 20661958

[B12] ACOG Committee Opinion. Number 326, December 2005 Inappropriate use of the terms fetal distress and birth asphyxia Obstet Gynecol 2005 106 6 1469 1470 10.1097/00006250-200512000-00056 16319282

[B13] Hankins GD Speer M Defining the pathogenesis and pathophysiology of neonatal encephalopathy and cerebral palsy Obstet Gynecol 2003 102 3 628 636 10.1016/S0029-7844(03)00574-X 12962954

[B14] Lin HC Sheen TC Tang CH Kao S Association between maternal age and the likelihood of a cesarean section: a population-based multivariate logistic regression analysis Acta Obstet Gynecol Scand 2004 83 12 1178 1183 15548152 10.1111/j.0001-6349.2004.00506.x

[B15] Lieberman E Lang JM Cohen AP Frigoletto FD Jr Acker D Rao R The association of fetal sex with the rate of cesarean section Am J Obstet Gynecol 1997 176 3 667 671 10.1016/S0002-9378(97)70567-2 9077626

[B16] Kessner DMSJ Kalk CE Schlesinger ER Infant death: an analysis by maternal risk and health care. In., edn 1973 Institute of Medicine and National Academy of Scientists: Washington, DC

[B17] Kotelchuck M An evaluation of the kessner adequacy of prenatal care index and a proposed adequacy of prenatal care utilization index Am J Public Health 1994 84 9 1414 1420 10.2105/AJPH.84.9.1414 8092364 PMC1615177

[B18] Malabarey OT Balayla J Abenhaim HA The effect of pelvic size on cesarean delivery rates: using adolescent maternal age as an unbiased proxy for pelvic size J Pediatr Adolesc Gynecol 2012 25 3 190 194 10.1016/j.jpag.2012.01.002 22578479

[B19] Volgyi E Tylavsky FA Xu L Lu J Wang Q Alen M Cheng S Bone and body segment lengthening and widening: a 7-year follow-up study in pubertal girls Bone 2010 47 4 773 782 10.1016/j.bone.2010.07.007 20637322

[B20] Leitch CR Walker JJ The rise in caesarean section rate: the same indications but a lower threshold Br J Obstet Gynaecol 1998 105 6 621 626 10.1111/j.1471-0528.1998.tb10176.x 9647152

[B21] Clark JF Westney LS Lawyer CJ Adolescent pregnancy: a 25-year review J Natl Med Assoc 1987 79 4 377 380 3586034 PMC2625495

[B22] Kozak LJ Weeks JD U.S. trends in obstetric procedures, 1990–2000 Birth 2002 29 3 157 161 10.1046/j.1523-536X.2002.00182.x 12153645

[B23] Lydon-Rochelle MT Holt VL Nelson JC Cardenas V Gardella C Easterling TR Callaghan WM Accuracy of reporting maternal in-hospital diagnoses and intrapartum procedures in Washington State linked birth records Paediatr Perinat Epidemiol 2005 19 6 460 471 10.1111/j.1365-3016.2005.00682.x 16269074

[B24] Frost F Starzyk P George S McLaughlin JF Birth complication reporting: the effect of birth certificate design Am J Public Health 1984 74 5 505 506 10.2105/AJPH.74.5.505 6711731 PMC1651619

[B25] American Academy of Pediatrics, Committee on Fetus and Newborn; American College of Obstetricians and Gynecologists, Committee on Obstetric Practice The Apgar Score Pediatrics 2006 117 1444 144716585348

